# The Brown-Séquard “Elixir”

**Published:** 1889-10

**Authors:** 


### Editorial.

### THE BROWN-SÉQUARD "ELIXIR."

The course of the experiments in the subcutaneous injection of testicular fluid, first suggested by M. Brown-Séquard before the Societé de Biologie of Paris in June last, has been such as to call for comment from scientific men in all quarters. As soon as the suggestion was made public in this country, a large number of physicians—many of whom, as results have shown, were wholly unfit, because of their lack of a knowledge of the importance of antisepsis, which should be observed whenever injections or incisions are to be made through the epidermis—hastened to perform the operation. In many instances urged on by patients, they rushed in where men of more extended experience would have hesitated to operate until they knew more of the methods adopted by Brown-Séquard in his experiments. Not only did incompetent men first take up the treatment, but they went into print, heralding their "expectations," which in many instances fell to the ground, their patients returning to them suffering from veritable septicæmia, as the natural result of the injection of putrescent matter. By so doing they have not only done themselves harm, but have been the means of causing injustice to be done a real scientific discovery.

The strangest part of the whole matter has been the position taken by many of the medical journals throughout the country, which in many instances have joined in the popular clamor against a treatment that had not had a fair trial. The results of the bungling, unscientific operations of wholly incompetent men have been used to decry the value of the real scientific experiments of a man whose many additions to our knowledge of scientific medicine should have entitled him to at least a respectful hearing. Let us review the history of the case, and learn the real claims made by M. Brown-Séquard. In the first place, he did not call his discovery an "elixir." That name was given to it by the secular press. He only claimed for the fluid obtained by crushing the testicles of healthy, vigorous animals a marked tonic effect, which seemed to be especially manifested in the cerebral and spinal centres. He stated very plainly that its effect was not lasting, disappearing in his case in about three weeks. All fair-minded medical men must admit that his *à priori* reasoning which led up to his experimentation was sound. The effeminacy of eunuchs is proverbial. The wide difference between the gelding and the stallion is also well known to horsemen. The marked debilitating effect of masturbation or sexual excess, when practised before the individual has fully matured, or when his powers are declining during old age, is also well known. The mental and physical depression dependent upon the loss of semen from any cause is out of all proportion to the effect produced by the nervous excitement accompanying such loss.

On the other hand, it is well known that abstinence in sexual intercourse gives increased strength and mental force. Athletes are very strict in regard to the matter of sexual intercourse while in training, and literary men often absent themselves from their wives when they have an especially hard task to perform. These well-known clinical facts led Brown-Séquard to the conclusion that semen possessed a marked dynamogenic power, and, when allowed to collect in the seminal vesicles, was absorbed into the system.

He did not attempt to say what the nature of the tonic principle was, but demonstrated, to his own satisfaction at least, that in cases of debility, especially that accompanying old age, as in his own case, its lack could be supplied by the injection of semen from healthy animals. He did not jump at these conclusions, but had for many years held these views, and had made experiments upon animals as early as 1875, and had stated his ideas upon the subject several years previously, as early as 1869, in a course of lectures before the Paris Faculty of Medicine. In discussing the influence possessed by several glands upon the nervous centres, he put forward the idea that if it were possible without danger to inject semen into the blood of old men, we should probably obtain manifestations of increased activity as regards the mental and the various physical powers.

The danger which at that time was considered certain from the injection of organic matter into the system no doubt prevented him from making the experiment then. Nevertheless, in 1875, he made some inoculations on dogs, which, while negative in most instances, yet were sufficiently successful to keep up his interest in the investigation. Finally, in 1888, he again began his experiments, regarding which he says:

"At the end of last year I made on two old male rabbits experiments which were repeated since on several others, with results leaving no doubt as regards both the innocuity of the process used and the good effects produced in all those animals. This having been ascertained, I resolved to make experiments on myself, which I thought would be far more decisive on man than on animals. The event has proved the correctness of that idea.

"Leaving aside and for future researches the questions relating to the substance or substances which, being formed by the testicles, give power to the nervous centres and other parts, I have made use, in subcutaneous injections, of a liquid containing a small quantity of water mixed with the three following parts: first, blood of the testicular veins; secondly, semen; and, thirdly, juice extracted from a testicle, crushed immediately after it has been taken from a dog or a guinea-pig."

"I have hitherto made ten subcutaneous injections of such a liquid,—two in my left arm, all the others in my lower limbs. The day after the first subcutaneous injection, and still more after the two succeeding ones, a radical change took place in me, and I had ample reason to say and to write that I had regained at least all the strength I possessed a good many years ago. Considerable laboratory work hardly tired me. To the great astonishment of my two principal assistants, Drs. D'Arsonval and Hénocque, and other persons, I was able to make experiments for several hours while standing up, feeling no need whatever to sit down. Still more: one day (the 23d of May), after three hours and a quarter of hard experimental labor in the standing attitude, I went home so little tired that after dinner I was able to go to work and to write for an hour and a half a part of a paper on a difficult subject. For more than twenty years I had never been able to do as much. From a natural impetuosity, and also to avoid losing time, I had, till I was sixty years old, the habit of ascending and descending stairs so rapidly that my movements were rather those of running than of walking. This had gradually changed, and I had come to move slowly up- and down-stairs, having to hold the banister in difficult staircases. After the second injection I found that I had fully regained my old powers, and returned to my previous habits in that respect."

Not only was his physical condition improved, but his capacity for mental labor was markedly increased.

To guard against the change of auto-suggestion, he had his limbs tested with a dynamometer for a week previous to the trial, and during the month following: the average number of kilogrammes moved by the flexors of the right forearm before the first injection was about 34½ (from 32 to 37), and after that injection 41 (from 39 to 44), the gain being from 6 to 7 kilogrammes. In that respect the forearm flexors reacquired, in a great measure, the strength they had when living in London (more than twenty-six years before).

Brown-Séquard's experiments have been to a certain extent confirmed by M. Variot, who made a communication to the Societé de Biologie on June 29. The patients chosen were debilitated men, aged fifty-four, fifty-six, and sixty-eight years respectively, and they were not informed of the nature of the treatment adopted. In all three cases the injections were followed by general nervous excitement, increased muscular power, and stimulation and regulation of digestion. M. Variot's observations dispose of the objection that the results observed by Brown-Séquard in himself were due to "suggestion." These experiments, together with some that have come under our notice, lead us to believe in the efficacy of the treatment. Two cases, among others which have been observed by us, have shown such marked benefit that we will relate their history.

One was that of a man past sixty years of age, whose occupation was of such a character that he was compelled to be on his feet much of the time during the day, and whose evenings were spent in casting up his accounts. During the summer he found himself so "run down," as he expressed it, that he would fall asleep over his accounts, and very much feared that he would have to give up his position. After one injection he was so much improved that he said he felt twenty years younger, and when asked whether he wanted a second, remarked that "so long as I feel as well as I do now there is no need of it."

The other was a man in the prime of life, who was engaged in constant mental work. For six weeks previous to the treatment he had been suffering from congestions of the pons and upper part of the spinal cord, brought on by overwork. He was completely incapacitated for mental work, but was unable to get away for a much-needed rest. He complained of great lassitude, especially marked at night, and a heavy, weary sensation at the base of the brain, with loss of the use of his right arm for a time Physical exertion, walking, relieved the congestion of the nervous centres, but did not give needed strength for mental labor. What was needed was complete rest, or a nervous tonic that would bridge over the present and allow nature to reassert herself, the patient being otherwise in good health. He had used the phosphates until they had apparently lost their efficacy, and then decided to try Brown-Séquard's "elixir." In this instance it did not show any stimulating effect worth noting. Three hours after the injection was made the pulse-beat had slightly increased; but the temperature remained stationary; there was slight exhilaration only, as shown by the increased tension of the pulse. On the second day he reported feeling considerably better, and on the third day resumed his ordinary literary occupation, with entire absence of the previous weariness complained of. Two weeks later he reported having felt a slight return of the old complaint, but only after an unusually hard day's labor, both mental and physical. His general condition was undoubtedly very much improved.

A physician here in the city has had similar results in several instances, but is withholding his report until there is a change in the attitude of the medical press towards the question, which up to the present has virtually classed all men as charlatans who have experimented with the "elixir." Appearances, however, point to a change of front. Daniel's *Medical Journal*, Austin, Texas, says, editorially, in the September number:

"In our last issue we said, 'To us there is something indescribably ludicrous in the idea, or association of ideas, that a general tonic or nerve stimulant was to be found in the testicles of a young animal.'

"Now we wish we hadn't said it. There are more strange things in chemistry than ever were dreamed of in our philosophy, Horatio,—and we have 'put our foot in it.' Well, there is consolation in the reflection that wiser men than we,—to wit, the erudite editor of the *Medical and Surgical Reporter*, and others,—who have ridiculed the idea, are in the same boat, and if we are laughed at, they will be also.

"It transpires that Parke, Davis & Co. have, with that intelligent zeal in the cause of new remedies which for years has characterized them, sought, found, and isolated the active principle of the 'juice' prepared according to Brown-Séquard's formula, and they recognize it as 'spermine'—an alkaloid first described by Charcot and Neumann.

"This alkaloid is found in the animal economy, elsewhere than in the testicles, and in the various excretions, especially in the sputa of consumption; in all wasting diseases it is excreted in abundance, and hence there is philosophy and good sense in the suggestion to replace it. Tests made with this alkaloid have proven it to possess decided stimulating effects upon the cerebro-spinal centres, and there is a possibility, after all, that it may be utilized as a remedy in exhaustion, etc. Moreover, Parke, Davis & Co. have prepared a salt of the alkaloid, the 'hydrochlorate of spermine,' for hypodermic use which is now on the market."

We may expect to hear from others in a similar strain ere long. This is not the first time a real discovery has been made the subject of ridicule. There are journals which even yet discredit the value of Koch's and Pasteur's discoveries. It has ever been thus, and perhaps always will be so. The world is most unfair, but the worst is that it too often cloaks its true inwardness under the plea of conservatism.

